# Nutritional Interventions for COVID-19: A Role for Carnosine?

**DOI:** 10.3390/nu13051463

**Published:** 2021-04-26

**Authors:** Jack Feehan, Maximilian de Courten, Vasso Apostolopoulos, Barbora de Courten

**Affiliations:** 1 Institute for Health and Sport, Victoria University, Melbourne, VIC 3011, Australia; jack.feehan@vu.edu.au; 2 Department of Medicine—Western Health, The University of Melbourne, Melbourne, VIC 3021, Australia; 3 Mitchell Institute for Education and Health Policy, Victoria University, Melbourne, VIC 3011, Australia; Maximilian.deCourten@vu.edu.au; 4 Department of Medicine, School of Clinical Sciences, Monash University, Clayton, VIC 3168, Australia

As COVID-19 continues to take an enormous toll on global health, the effort to find effective preventive and treatment strategies has been unparalleled in recent history [1]. While the rapid rollout of vaccines is heartening, limited supply, logistical hurdles, enormous demand, and the rise of new variants of the virus will make population-scale vaccination a challenging target to meet, particularly in lower socioeconomic areas of the world [2]. This makes cheap, safe, and effective measures to limit the impact of COVID-19 of vital importance, and in this, nutritional interventions are particularly attractive as they are readily scalable. While some nutrients have garnered widespread attention for their potential use in preventing or managing COVID-19, such as vitamins B, C, and D [3,4], there are many others with powerful health-promoting effects that may have clinical utility. Carnosine is one such nutritional supplement, which could protect against some of the detrimental effects of SARS-CoV-2 infection.

Carnosine is an over-the-counter nutritional supplement with a wide array of beneficial physiological effects in vivo [5,6]. Carnosine is synthesized from beta-alanine and histidine and is concentrated particularly in muscle, cardiac, and brain tissues. Carnosine is often regarded as a geroprotector, offering a range of benefits thought to improve age-related chronic diseases, longevity, and “health-span”. Supplementation with carnosine is common in athletes, due to its performance-enhancing properties. However, it is cheaper to use β-alanine, which is also the rate-limiting factor in carnosine synthesis [7]. Importantly, carnosine has strong anti-inflammatory, antioxidant, and anti-glycating effects, all important factors in the initiation and progression of many chronic conditions associated with advanced age, including diabetes and its complications, cardiovascular disease, and neurodegenerative diseases [8]. While anti-inflammatory, anti-oxidative, and anti-glycating effects make carnosine a worthy target for COVID-19 (Figure 1), more recently, carnosine has also been shown to have anti-viral properties [9,10,11,12]. The flaviviruses Zika virus and dengue virus have re-emerged, causing significant outbreaks. Although the pathophysiology of these viruses is understood, no vaccines or anti-viral drugs have been approved. Through *in silico* studies, it was noted that carnosine could interact with viral proteins, and in *in vitro* studies carnosine was able to inhibit dengue virus and Zika virus infection and replication in human liver cells [12]. The ability of carnosine to decrease nitric oxide and neutrophil influx into the upper respiratory tract has been noted to be important in controlling the initial stages of influenza A infection [10,11]. In mice, carnosine decreases mortality and pathological lung changes following infection with H9N2 swine influenza virus [9]. Due to all of these benefits, carnosine could also have a role in protecting patients against COVID-19.

COVID-19 is known to cause significant immune dysregulation, with a host of knock-on effects in patients. The immune system of a patient with COVID-19 must achieve a tight balance between achieving a sufficient immune response to repel the pathogen, without initiating an excessive inflammatory response. This excessive response, known as a cytokine storm, is central to many of the most damaging and potentially life-threatening consequences of COVID-19 [13]. This cytokine storm is characterized by a significant upregulation of inflammatory cytokines, notably interleukin (IL)-1, IL-6, and tumor necrosis factor alpha, as well as being associated with a high neutrophil to lymphocyte ratio [13]. Carnosine has been demonstrated to ameliorate these markers of immune hyperactivity in models of lipopolysaccharide and bleomycin-induced lung injury [14,15], indicating that it may prevent the excessive immune response in patients with COVID-19. This is likely to occur through its anti-inflammatory and anti-oxidant actions [16], as COVID-19 patients have been shown to be under significant inflammation and oxidative stress, which has been suggested to influence the hyperinflammatory response [17]. The cytokine storm of COVID-19 can also lead to acute respiratory distress syndrome (ARDS). Patients with ARDS due to COVID-19 have a higher mortality rate than those without and are commonly not responsive to ventilation and other supportive treatments [18]. Carnosine has been shown to protect against lung injury associated with ARDS, through its powerful antioxidant effects, reducing reactive oxygen species -mediated toxicity to the lung cells in animal models of lipopolysaccharide-induced pulmonary injury [14] and H9N2 swine influenza [9]. The combined anti-inflammatory and anti-oxidative effects of carnosine make it particularly suited to be considered as supportive treatment in patients with COVID-19.

While the anti-inflammatory and anti-oxidant properties of carnosine are well established, there are some other potential avenues by which it may aid in the treatment of COVID-19. While originally thought to be a primary respiratory pathology, it is now known that COVID-19 is a system-wide disease, leading to multi-organ failure, and cerebrovascular events. This is at least partially due to the increase in coagulation associated with the infection, which leads to microvascular damage, and stroke [19]. It has been proposed that the anti-oxidant and anti-glycating properties of carnosine may provide protection from this hypercoagulability, particularly in patients with altered glucose metabolism, who are known to have increased COVID-19 morbidity and mortality [16,17]. Others have suggested that carnosine may in fact prevent binding and internalization of SARS-CoV-2, potentially reducing both the incidence and severity of disease [20]. The SARS-CoV-2 spike protein has high binding affinity for angiotensin-converting enzyme 2 (ACE2), using this as a means of attachment and entry to the host cell [21]. *In silico* modelling of known pharmaceutical and therapeutic compounds revealed that carnosine is also able to bind to ACE2 as an inhibitor, potentially competing with the SARS-CoV-2 spike protein and reducing effective entry to host cells [20].

A new and concerning development in the management of patients with COVID-19 is the newly understood syndrome of “long COVID” in which symptoms develop and evolve over weeks and months following the infection [22]. Symptoms of long COVID are varied between individuals, but commonly include fatigue, physical pain, reduced exercise tolerance, dyspnea, gastrointestinal symptoms, headaches, memory and concentration difficulties, and psychological disturbance [22,23]. While research into the underlying cause of long COVID syndrome is in its infancy, it is currently thought to be the result of widespread, chronic multi-system inflammation, secondary to significant inflammatory activation, and cytokine storm [24,25]. This inflammation is thought to affect the lungs and cardiovascular, central nervous, and gastrointestinal systems [25,26,27], however, many questions remain. As patients with long COVID report significant decreases in quality of life [22], identifying strategies to manage it are critical. Given its inflammatory and anti-oxidative properties as well as its effects on exercise performance [28] and cardiac and cognitive function [3], carnosine supplementation could prevent or at least reduce symptoms related to long COVID. In addition, if the cytokine storm due to COVID-19 is rapidly suppressed, it is possible that ongoing symptoms may be decreased, or avoided entirely. Carnosine should therefore be evaluated for use in these patients.

While carnosine supplementation may have a beneficial impact alone, there is also potential for its use alongside other nutritional interventions in patients with COVID-19. Several other nutrients have been investigated for use in patients infected with COVID-19, with potentially synergistic immune effects. Vitamins D, C, and B are all known to be immunomodulatory, and have been suggested to have potential in dampening the aggressive immune response and cytokine storm associated with COVID-19 [3,4]. Other trace minerals such as zinc and selenium, as well as omega-3 fatty acids, have also been shown to have some benefits which may improve the outcomes of patients with COVID-19, improving outcomes in respiratory infection, which has led many to recommend their use [29]. Interestingly, others have recommended pre- and pro-biotic interventions, as they also improve respiratory symptoms [30]. A “multi-nutrient” intervention, consisting of carnosine, alongside these additional immune-enhancing vitamins and minerals, could be used to enhance patient outcomes.

While research into these potential effects is preliminary, the role of carnosine in patients with COVID-19 is promising and therefore there is a need for trials to test the efficacy of carnosine for prevention, as a supportive measure during the infection as well as in long COVID (Figure 1). Given the high tolerability and absence of significant side effects, alongside its anti-inflammatory and an antioxidant effects, it could provide a low-cost, scalable solution for this indication. This is particularly noteworthy given carnosine’s well-known benefits in patient demographics who are at increased risk from COVID-19, including the elderly and those living with diabetes.

## Figures and Tables

**Figure 1:**
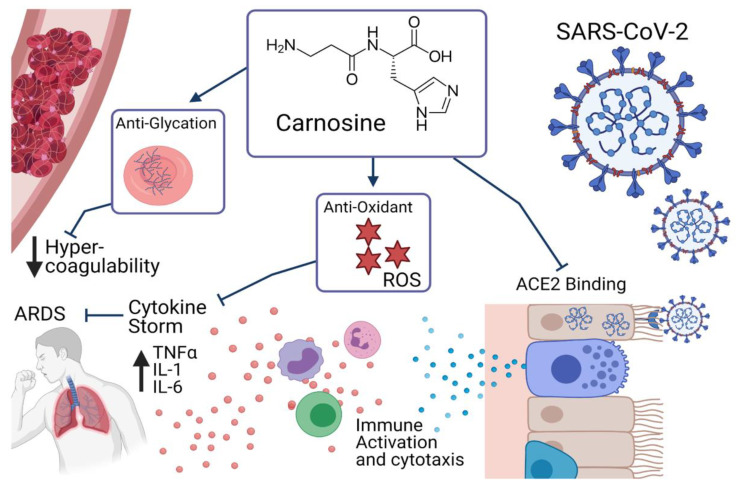
Effects of carnosine in relation to COVID-19.
